# Healthcare Workers’ Acceptance of COVID-19 Vaccination in Russia

**DOI:** 10.3390/ijerph19074136

**Published:** 2022-03-31

**Authors:** Nikolay I. Briko, Vladimir A. Korshunov, Alla Ya Mindlina, Roman V. Polibin, Maksim O. Antipov, Alexey I. Brazhnikov, Yurii E. Vyazovichenko, Ekaterina V. Glushkova, Kirill S. Lomonosov, Alena V. Lomonosova, Platon D. Lopukhov, Artem A. Pozdnyakov, Tatiana S. Saltykova, Nikolay V. Torchinsky, Natalia N. Tsapkova, Olga P. Chernyavskaya, Arseny V. Shamis

**Affiliations:** Department of Epidemiology and Evidence-based Medicine, I.M. Sechenov First Moscow State Medical University (Sechenov University), 119991 Moscow, Russia; briko_n_i@staff.sechenov.ru (N.I.B.); mindlina_a_ya@staff.sechenov.ru (A.Y.M.); polibin_r_v@staff.sechenov.ru (R.V.P.); antipov_m_o@staff.sechenov.ru (M.O.A.); brazhnikov_a_yu@staff.sechenov.ru (A.I.B.); vyazovichenko_yu_e@staff.sechenov.ru (Y.E.V.); glushkova_e_v@staff.sechenov.ru (E.V.G.); lomonosov_k_s@staff.sechenov.ru (K.S.L.); lomonosova_a_v@staff.sechenov.ru (A.V.L.); lopukhov_p_d@staff.sechenov.ru (P.D.L.); pozdnyakov_a_a@staff.sechenov.ru (A.A.P.); saltykova_t_s@staff.sechenov.ru (T.S.S.); torchinskiy_n_v@staff.sechenov.ru (N.V.T.); tsapkova_n_n@staff.sechenov.ru (N.N.T.); chernyavskaya_o_p@staff.sechenov.ru (O.P.C.); senior.shamis@yandex.ru (A.V.S.)

**Keywords:** Russia, healthcare workers, SARS-CoV-2, vaccine acceptance, vaccine hesitancy

## Abstract

During the COVID-19 pandemic, the problem of the population’s adherence to vaccination has become significantly aggravated around the world. This study is aimed at evaluating healthcare workers’ (HCWs) acceptance of COVID-19 vaccination in Russia. A cross-sectional multicenter study was carried out by interviewing HCWs in Russia using an electronic questionnaire and snowball sampling. The analysis included 85,216 questionnaires from 81 out of 85 regions of Russia. Statistical analysis was performed using SPSS v.22. The results indicated that 35.0% (CI 95%, 34.7–35.3) of HCWs were ready to get COVID-19 vaccination. The acceptance level was 42.4% (41.8–42.9) for all physicians and 31.3% (30.9–31.6) for nursing staff. A total of 29.4% (29.1–29.7) of HCWs were willing to recommend COVID-19 vaccination to patients: 38.5% (38.0–39.1) of physicians, and 24.7% (24.4–25.1) of nursing staff. Acceptance of COVID-19 vaccination is higher among HCWs dealing with infectious diseases and involved in vaccination. The low acceptance of HCWs toward vaccination against COVID-19 can be explained by the low level of awareness of HCWs in these issues. Additional educational programs are needed for HCWs, both for physicians and nurses, using all possible forms and methods of education.

## 1. Introduction

During the COVID-19 pandemic, caused by the SARS-CoV-2 virus, the problem of the population’s adherence to vaccination has become significantly aggravated around the world [1,2,3]. The situation across countries is unequal both in terms of vaccination coverage against coronavirus infection and adherence to vaccination [4]. Acceptance rates in the total population vary from 23.6% in Kuwait and 28.4% in Jordan to more than 90% in Ecuador, Malaysia, Indonesia and China. Russia is among the countries with the lowest levels of COVID-19 vaccine acceptance (54.9%) [1].

Healthcare workers play the most important role in promoting vaccination to the public, and influence people’s decision on whether to be vaccinated or not [5]. Additionally, the vaccination of healthcare workers against COVID-19 is considered to be a priority [6]. It was shown that the prevalence of COVID-19 vaccination hesitancy worldwide in healthcare workers ranged from 4.3 to 72% [2]. Some of the highest healthcare workers’ acceptance rates were observed in Germany (91.7%) [7] and Canada (80.9%) [8]. The acceptance rate was 64.7% for doctors and 34.5% for nurses in Poland [9]. In the United Arab Emirates, 58% of HCWs were willing to take the vaccine and give it to their family [10]. In a study of HCW in the United States, 36% of respondents were willing to take the vaccine as soon as it became available. [11] However, data on the acceptance rate of COVID-19 vaccination among HCWs in Russia were not available before the present study.

Russia was the first country to register a vaccine against coronavirus disease 2019 (COVID-19), on 11 August 2020, for emergency use in the pandemic according to Government Decree No.441 of 03 April 2020, which introduced a simplified registration procedure for medical products during the pandemic. The first confirmed case of COVID-19 in Russia was identified on 3 March 2020. After 2 years of the pandemic (as of 3 March 2022) a total of 16,445,802 cases of COVID-19 (11,271 per 100,000) and 346,967 deaths (237.8 per 100,000) due to COVID 19 have occurred in Russia. During this period, five significant rises in incidence (“waves”) can be identified.

Since December 2020, a voluntary vaccination campaign against the infection caused by SARS-CoV-2 has begun in Russia [12], and in January 2021 a mass vaccination campaign was started, which allowed all citizens to be vaccinated, regardless of their risk group and profession [13]. At that time, a vector vaccine Gam-COVID-Vac (“Sputnik V”) was available in Russia [14]. At the same time, for the past few years there has been a trend towards decreased compliance to preventive vaccination both globally and in Russia. The distrust in vaccines and rise of the anti-vaccination movement in 2019 were included in the top ten threats to global health [15]. In medical workers, who are generally negative about vaccination, we can expect a lower level of acceptance of COVID-19 vaccination. The worsening of the epidemiological situation associated with the spread of the new coronavirus infection further promoted public discussions on vaccination in the media. This can also explain the change in attitude toward this issue, both in the professional community and the general public.

Thus, we have conducted a study to evaluate the overall vaccination acceptance of different healthcare professionals and changes in the vaccination compliance in view of the COVID-19 pandemic. This article reflects the evaluation of healthcare workers’ acceptance to COVID-19 vaccination.

## 2. Materials and Methods

We carried out a cross-sectional study to evaluate the attitude of physicians and nurses to the COVID-19 vaccination. The data was collected in January 2021 (15 January 2021–31 January 2021). Materials for the study were collected prior to the publication of the results of a Phase III clinical study of Sputnik V vaccine in Russia in Lancet [16]. The study population includes healthcare workers from different types of healthcare facilities (outpatient, inpatient, etc.) aged over 18, who live in the Russian Federation and have been working in the healthcare system for at least one month before the start of the study. Students or medical residents working in healthcare organizations were not included in the study. We also excluded the results from the responses of non-medical staff in healthcare facilities from this study. Informed consent was obtained before subject inclusion in the study.

The study was conducted in accordance with the Declaration of Helsinki and requirements of Federal Law No. 152 of the Russian Federation “On personal data” and approved by the Ethics Committee of I.M. Sechenov First Moscow State Medical University (protocol code № 01-21 date of approval 12 January 2021).

### 2.1. Data Collection Procedure

In our study, we used the snowball sampling procedure. We asked the National Association of the Specialists in Control of Health Care-Associated Infections (NP “NASCI”) to send the link to the electronic questionnaire with the invitation to take part in the study [17]. Invitations were sent in informational letters to healthcare personnel, such as regional chief specialists, healthcare department officers, and chief physicians, in each of the 85 regions of Russia.

We received a total of 89,432 filled-in questionnaires, and responses from 85,216 healthcare workers who met our inclusion criteria, filled in the questionnaire correctly, and were thus included in the study.

The information about the number of healthcare workers is freely available from the Federal Research Institute for Health Organization and Informatics of Ministry of Health of the Russian Federation [18]. The number of healthcare organizations was taken from the website (Available online: https://rosstat.gov.ru, accessed on 19 March 2022).

### 2.2. Questionnaire

We developed the questionnaire using data from the literature. All questions from the survey were reviewed by experts in the field of social sciences, preventive immunization, and epidemiology. Additionally, the survey was preliminarily tested for clarity, time needed for its completion, and validity. The testing process involved 50 healthcare workers and resulted in the introduction of insignificant changes to the questionnaire.

The questionnaire included demographic data (age, gender, place of work (region), location of healthcare facility (rural/urban area), level of education (physician/nurses), length of employment, questions concerning vaccination (attitude to vaccination in general and to the COVID-19 vaccination in particular, involvement in vaccination)), as well as healthcare workers’ specialization. To evaluate healthcare workers’ attitude to vaccination in general, we used a 10-point Likert scale. When asked about the attitudes towards vaccination, the respondent had to indicate a value from 1 to 10, where 1 is a completely negative attitude, 10 is a completely positive attitude, and 5 is neutral. The questionnaire provided instructions for answering this question.

Regarding COVID-19 vaccination, two main questions were asked, namely, “Are you willing to be vaccinated against COVID-19?” (Yes/No) and “Are you willing to promote COVID-19 vaccination to your patients, acquaintances, and relatives?” (Yes/No/Not sure).

### 2.3. Study Conduct

Information was collected online using the questionnaire. We did not publish the questionnaire online for open access but distributed it via the local healthcare authorities (chief regional specialists, healthcare department officers, chief physicians). The questionnaire was accessed via invitations sent in the information letters.

The study was anonymous; we did not ask for or collect the name, surname, institution name and any other information that allows to identify an individual or respondent. The completed forms were not available to institution administration or local medical authorities.

### 2.4. Statistical Analysis

Statistical analysis was performed using IBM SPSS Statistics v.22. Data analysis was carried out using descriptive statistics. For quantitative variables, the methods included the calculation of mean values, variance, standard deviation, standard error of the mean, confidence interval, median values, 25th and 75th percentiles, and interquartile range. The qualitative variables were checked for normality of distribution using Lilliefors test. Statistical significance of differences of non-normally distributed quantitative variables was determined using Mann–Whitney U test with an assumed *p*-value < 0.05. Statistical associations between two non-normally distributed quantitative variables were assessed using Kendall’s tau coefficient.

For the description of data, we used mean values, standard deviation, and standard error of the mean for normally distributed quantitative variables, and median values and interquartile range for non-normally distributed variables.

For qualitative variables, we used methods of descriptive statistics such as the calculation of the proportion, standard error of the proportion, and 95% confidence interval. The statistical significance of differences in the groups of qualitative variables was determined using the chi-square test with an assumed *p*-value < 0.05. Relationships between qualitative variables were assessed using Cramer’s V coefficient. Data for qualitative variables are presented as proportions and 95% confidence interval for the proportion.

## 3. Results

### 3.1. Characteristics of Participants

The study included healthcare workers of different age groups. The median age was 42 years (IQR 32–51). The youngest participant was 18 years of age, and the eldest was 89 years of age. The median length of employment was 19 years (IQR 8–29). The study included both young healthcare workers (6.6% had worked in the healthcare system for less than a year) and specialists with extensive professional background (4.3% had worked in the healthcare system for 40 or more years). Most of the respondents were female (90.2%) which reflects the overall predominance of women in healthcare. Almost half of the respondents worked in outpatient facilities (48.5%), one-third in inpatient facilities, and slightly over 15% in other healthcare organizations. The majority of the respondents worked in urban healthcare facilities (Table 1).

Participation in the survey was anonymous and voluntary; therefore, not everyone who had received the invitation participated in the study. Healthcare workers in 81 out of 85 constituent entities of the Russian Federation took part in the study. Nevertheless, the study encompassed all the federal districts of Russia. A total of 5.2% of healthcare workers of the Russian Federation were interviewed. A slightly higher coverage was achieved in the Far Eastern and North-Western federal districts (8.4% and 8.2%, respectively). The Southern federal district was the least represented in this study. Nonetheless, the amount of data obtained allows us to make conclusions on the country level, and on certain regional aspects.

Out of all the interviewed healthcare workers, 33.5% were physicians and 66.5% were nursing staff. This distribution also reflects the overall ratio of physicians and nurses in Russia. Apart from clinical physicians, the study included healthcare management personnel (chief physicians and their deputies) and hospital epidemiologists. In the Russian healthcare system, hospital epidemiologists are physicians having a medical degree who provide infection control in a healthcare facility to prevent healthcare-associated infections (Table 2).

### 3.2. Involvment in Vaccination

Out of the interviewed healthcare workers, 37.9% of the respondents (29.8% of nursing staff and 54.1% of physicians) were involved in vaccination (subjects’ selection for vaccination, patient consultation, injection of vaccines). These specialists were assigned to a separate category: “Involved in vaccination process”, which included all of the previously mentioned variants.

The following healthcare specialists were mostly involved in vaccination: primary care physicians (primary care physicians, general practitioners, and family doctors were included in one group)—87.7%, pediatricians—84%, and a number of specialist physicians such as allergologists and immunologists (77%), pulmonologists (70.4%), and gynecologists (69%). Clinical physicians (surgeons, ENT specialists, dermatologists, etc.), who are much less involved in vaccination, were grouped under one category: other clinical physicians.

### 3.3. Willingness to Be Vaccinated

**Figure 1:**
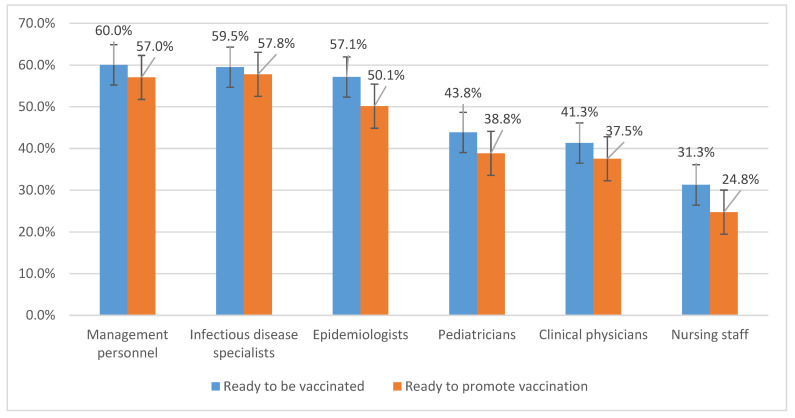
The adherence of health care providers to vaccination against COVID-19.

The study results show that in the cases where vaccination was indicated, 35.0% (CI 95%, 34.7–35.3) of the interviewed healthcare workers were ready to be vaccinated. This proportion was higher for physicians as compared to nurses (42.4% and 31.3%, respectively). At the same time, the highest proportion of subjects ready to be vaccinated was for management personnel (60.0%; 57–63.1), infectious disease specialists (59.5%; 55.5–63.5), epidemiologists (57.1%; 53.6–60.7), and pediatricians (43.8%; 42.6–45.1). For other clinical physicians (primary care physicians, specialist physicians) this number was lower (41.3%; 40.7–41.9) (Figure 1).

The detailed distribution of responses is presented in Appendix A (Table A1 and Table A2). Among clinical physicians (except for infectious disease specialists and pediatricians), no statistically significant differences for this parameter have been found.

Healthcare workers in older age groups are more compliant to vaccination against COVID-19. Thus, the proportion of healthcare workers ready to be vaccinated varies from 24.2% (23.6–24.9) in the age group of 20–30 years to 45.8% (44.5–47.1) in the age group of 61–70 years with no significant differences in the mean age between healthcare workers of different specialties. A similar trend is observed in terms of the length of employment; however, this criterion cannot be considered an independent factor because it is directly related to the respondent’s age. For male healthcare workers, the proportion of respondents ready to be vaccinated is higher than in females: 43.2% (42.2–44.3) and 33.8% (33.5–34.2), respectively. These differences are consistent among physicians of all specialties and nursing staff.

The personnel of emergency and immediate care facilities are least compliant with COVID-19 vaccination (28.3%; 26.9–29.6), whereas the outpatient healthcare workers demonstrate the highest vaccination acceptance (37.4%; 36.9–37.9). The percentages of the personnel in other types of healthcare facilities were somewhere in between. At the same time, these differences can be attributed to the different ratios of physicians and nurses in different types of facilities. Thus, a separate analysis of healthcare workers’ categories showed no significant differences between the types of organizations. Acceptance of COVID-19 vaccination was higher for the healthcare workers in rural areas compared to the healthcare personnel in urban areas: 49.5% (39.6–41.4) and 33.9% (33.5–34.2), respectively.

For healthcare workers involved in vaccination, the proportion of those ready to be vaccinated against COVID-19 was significantly higher compared to those not involved in vaccination: 42.3% (41.7–42.8) and 30.5% (30.1–30.9), respectively. This is consistent with both nurses and clinical physicians, including all specialist physicians and excluding infectious disease specialists. Among infectious disease specialists, there are no significant differences in the proportion of those ready to be vaccinated: 56.2% (49.6–62.8) for those not involved in vaccination and 61.4% (56.4–66.4) for those involved in vaccination. This is consistent with the trend observed for the healthcare workers of other specialties.

It should be noted that the most important factor determining the willingness of healthcare workers to be vaccinated against COVID-19 is their personal attitude to vaccination. Thus, the proportion of healthcare workers ready to be vaccinated was 49.3% (48.9–49.8) for those with a positive attitude to vaccination, 19% (18.4–19.5) for those who are hesitant and 7.1% (6.6–7.5) for those with a negative attitude.

The differences in the willingness to be vaccinated between nursing staff and physicians who are in favor of vaccination were not significant: 52.4% (51.7–53.1) of physicians and 47.2% (46.6–47.7) of nurses were ready to be vaccinated. Nevertheless, the proportion of those willing to be vaccinated did not reach 100% in any category of healthcare workers, even among those with a positive attitude to vaccination. The percentage was the highest for healthcare management personnel and epidemiologists: 70.4% (67.2–73.7) and 62.8% (59.1–66.5), respectively. For clinical physicians it was the highest among infectious disease specialists: 69.3% (65.1–73.5).

The level of public trust in vaccination largely depends on the healthcare workers’ compliance and their willingness to promote vaccination, both at work (to patients) and outside work (to relatives, friends, and acquaintances). The study results show that the healthcare workers’ responses to this question can be divided into three groups similar in proportion: one-third (29.4% (29.1–29.7)) of healthcare workers were ready to promote vaccination, 33.8% (33.5–34.1) responded negatively, and 36.8% (36.5–37.2) were hesitant.

### 3.4. Willingness to Promote Vaccination

**Figure 2:**
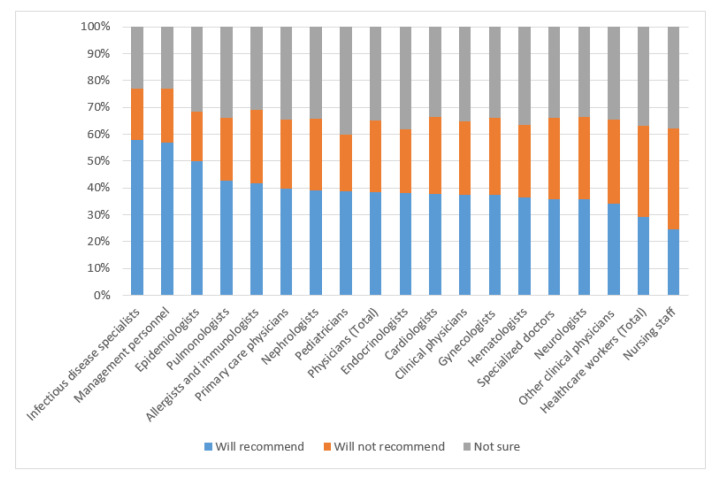
Willingness to promote vaccination against COVID-19.

As with the willingness to be vaccinated mentioned above, the percentage of healthcare workers willing to promote vaccination was higher for physicians (38.5% (38.0–39.1)) than for nurses (24.7% (24.4–25.1)), and for specialist physicians this proportion was 37.5% (37.0–38.1) (Figure 2).

This parameter was the highest for healthcare management personnel (57.0%; 53.9–60.2), epidemiologists (50.1%; 46.6–53.7), and infectious disease specialists (57.8%; 53.8–61.8). There were no considerable differences among specialist physicians (except for infectious disease specialists).

The detailed distribution of responses is presented in Appendix A (Table A3 and Table A4). The main predictors of the willingness to promote vaccination, as well as to be vaccinated, included male gender, older age (which corresponds to the length of employment), work in outpatient facilities, and work in rural areas. For federal districts, the percentage was the lowest in the North Caucasian district (21.3%; 20.0–22.6) and the highest in the Far Eastern (35.5%; 34.4–36.6) federal district.

Healthcare workers involved in vaccination expressed more willingness to promote vaccination against COVID-19 (37.5%; 37–38.1) as compared to those not involved in vaccination (24.4%; 24–24.7). For the respondents with a positive attitude to vaccination only 42.6% (42.2–43.1) replied “yes” to this question, 20.9% (20.5–21.3) replied “no”, and 36.5% (36–36.9) were hesitant.

## 4. Discussion

Comparing our results with similar research it should be noted that acceptance level in COVID-19 vaccine among HCWs in Russia (35.0%) is much less than in other countries. The level of willingness to have the COVID-19 vaccine was 50.52% in Saudi Arabia [19]. A cross-sectional study in China shows that 76.63% of participants (nurses, clinicians, administrative staff) declared they would accept the vaccine [20]. In a study among HCW in the United States, 36% of respondents were willing to take the vaccine as soon as it became available while 56% were not sure or would wait to review more data. Only 8% of HCWs did not plan to get vaccine [11]. The highest overall acceptance of the SARS-CoV-2 vaccine in HCWs was shown in Canada (80%) [8] and Eastern Cape, South Africa (90.1%) [21]. All of these studies were carried out in similar period to ours (November 2020–January 2021). A systematic review of global COVID-19 vaccine acceptance, published in January 2022 showed that the general healthcare workers (HCWs) in China (86.20%) and nurses in Italy (91.50%) had the highest acceptance rates, whereas HCWs in the Democratic Republic of Congo had the lowest acceptance (27.70%) [1].

A systematic review of the general population in 33 different countries also showed Russia among those with the lowest COVID-19 vaccine acceptance rates [3].

The HCWs perceived significantly higher susceptibility and severity of the COVID-19 infection compared to the general population [22]. A higher level acceptance of the COVID-19 vaccine was connected with male sex, older age, total vaccine acceptance, and higher levels of education (physician vs nurses) [2,8,11,19,21,23]. We found the same relationship in our study. Additionally, we have shown that the acceptance rate is higher for HCWs, management personnel in vaccination procedures, and staff dealing with infectious diseases. In some studies, it was also shown that vaccine acceptance increased with increasing income level [11], perceiving a high risk of infection, and believing that the COVID-19 vaccine should be compulsory for all citizens [8,19]. There is evidence that healthcare workers working with COVID-19 patients are more adhere to vaccination [2,8].

We did not include questions about why (for what reasons) healthcare workers do not accept vaccines in our study. However, in conversations with doctors and nurses, we heard mostly about safety concerns and insufficient research information about the COVID-19 vaccine. These same questions were asked by non-medical professionals. In the literature, the main reasons of low vaccination acceptance are concerns about vaccine safety, efficacy, and potential side effects and low levels of education and awareness [1,2].

Additionally, we did not re-measure vaccination adherence after the first survey. According to a systematic review of vaccine acceptance rates (Sallam, M., 2021) in countries with multiple surveys over time, the following changes in COVID-19 vaccine acceptance rates were observed. In the UK, the vaccine acceptance rate was 79.0% in April 2020, 83.0% in May 2020, 71.5% in June 2020, 64.0% in July 2020, and 71.7% in September/October 2020. In France, the vaccine acceptance rate ranged from 62.0% to 77.1% in March/April 2020 and was 58.9% in June 2020. In Italy, the vaccine acceptance rate was 77.3% in April, 70.8% in June, and it reached 53.7% in September 2020 [3]. In the US, HCWs acceptance of COVID-19 vaccine has also varied. In October–November 2020, approximately one-third of the surveyed population of HCWs were ready to take the vaccine as soon as it became available. In subsequent studies, vaccine acceptance has been reported in various healthcare systems at 55.3–57.5% in December 2020, 86% in January 2021, and 84.6% in February 2021 [24].

The relatively poor compliance to vaccination of healthcare workers revealed during the study may contribute to the current situation in the COVID-19 vaccination campaign in Russia. A comparison between the results of the COVID-19 vaccination campaign in Russia and the rest of the world for more than half a year shows much lower vaccination coverage in Russia, despite the availability of vaccines to the general public and wide media coverage of vaccination [4]. Some significant differences between the healthcare workers’ categories may serve as the basis for determination of main predictors of the willingness to be vaccinated and promote vaccination.

The higher level of COVID-19 vaccine acceptance in HCWs dealing with infectious diseases (infectious disease specialists, epidemiologists), as well as healthcare workers involved in vaccination can be explained by their better awareness of the subject. We suppose that by working in this field they are better informed about vaccine safety, efficacy, and mechanisms of action, and they are less susceptible to the influence of anti-vaccination communities.

As expected, healthcare workers with an overall positive attitude to vaccination are more willing to be vaccinated and promote vaccination against COVID-19. However, the fact that even within this cohort of healthcare workers, who are most compliant to vaccination, the coverage has not reached 100% demonstrates that healthcare workers have some doubts on this subject. In this context, it should be noted that our study has some limitations as the materials for the study were collected before the publication of data of the Phase III clinical study of Sputnik V vaccine. This publication might have a certain impact on the acceptance of healthcare workers of COVID-19 vaccination.

The nursing staff demonstrated a significantly lower vaccination acceptance overall and against the coronavirus infection in particular compared to physicians (Table A5 and Table A6). The reason for such a low compliance of nurses is a debatable issue. It may be related to a much less involvement of these workers in vaccination or their poor interest in the subject. However, it should be noted that their patients, family members and acquaintances may ask them for consultation. In this context, the problem of nursing staff vaccination acceptance requires further investigation.

Healthcare workers of older age who have been working in the healthcare system longer are generally more compliant to vaccination against COVID-19, which can be attributed to the higher risks of this infection in older age groups.

## 5. Conclusions

The results of our study conducted among healthcare workers in the Russian Federation show that, provided vaccination is indicated, 35.0% (CI 95%; 34.7–35.3) of healthcare workers are ready to be vaccinated against COVID-19. This proportion was higher for physicians than for nurses: 42.4% (41.8–42.9) vs 31.3% (30.9–31.6), respectively. At the same time, the highest percentage of healthcare workers willing to be vaccinated is observed among healthcare management personnel (heads of healthcare organizations and their deputies) (60.0%; 57–63.1), epidemiologists (57.1%; 53.6–60.7), infectious disease specialists (59.5%; 55.5–63.5), and pediatricians (43.8%; range 42.6–45.1).

The percentage of healthcare workers willing to promote COVID-19 vaccination was 29.4% (29.1–29.7). A total of 38.5% (38.0–39.1) of physicians were willing to promote vaccination as opposed to 24.7% (24.4–25.1) are nurses. The highest percentage of healthcare workers willing to promote vaccination was among healthcare management personnel (57.0%; 53.9–60.2), epidemiologists (50.1%; 46.6–53.7), and infectious disease specialists (57.8%; 53.8–61.8). There were no significant differences among specialist physicians (except for infectious disease specialists).

The main predictors of the willingness to be vaccinated and to promote vaccination against the coronavirus infection were the overall attitude to vaccination and involvement in vaccination. The acceptance of COVID-19 vaccination was higher among male healthcare workers, those of older age, and those working in outpatient facilities and in rural areas.

In this regard, additional educational programs are needed for medical workers, both with higher and secondary medical education, using all possible forms and methods of education. In recent months, such work has been carried out in Russia, which contributes to a gradual increase in vaccination coverage against coronavirus infection.

## Figures and Tables

**Table 1:** Demographic characteristics.

Variable	N = 85218
**Age**	
18–30 years	17,548 (20.7%)
31–40 years	21,125 (24.9%)
41–50 years	23,779 (28.0%)
51–60 years	16,681 (19.7%)
61–70 years	5709 (6.7%)
>70 years	376 (0.4%)
**Gender**	
Male	8322 (9.8%)
Female	76,896 (90.2%)
**Place of work**	
Outpatient polyclinic institutions	41,316 (48.5%)
Inpatient medical institutions	31,210 (36.6%)
Dispensaries	4404 (5.2%)
Women’s consultations, Maternity hospitals	3647 (4.3%)
Ambulance and emergency care facilities	4100 (4.8%)
Spa facilities, preventive healthcare facilities	541 (0.6%)
**Healthcare facility in**	
Urban area	73,694 (86.5%)
Rural area	11,524 (13.5%)
**Federal district**	
Central FD	24,347 (28.6%)
Northwestern FD	12,903 (15.1%)
Volga FD	12,606 (14.8%)
Southern FD	2736 (3.2%)
North Caucasian FD	3902 (4.6%)
Ural FD	6694 (7.9%)
Siberian FD	14,214 (16.7%)
Far Eastern FD	7814 (9.2%)
Russian Federation (Total)	85,218 (100%)
**Proportion of surveyed physicians from the total number of doctors in the federal district**	
Central FD	6.5%
Northwestern FD	8.2%
Volga FD	3.6%
Southern FD	1.9%
North Caucasian FD	4.1%
Ural FD	4.1%
Siberian FD	5.7%
Far Eastern FD	8.4%
Russian Federation (Total)	5.2%

**Table 2:** Surveyed healthcare workers by specialty and their involvement in vaccination procedures.

	Surveyed		Involved in Vaccination Process (among Surveyed by Specialty)	
	Frequency	Percentage	Frequency	Percentage
**Total**	**85,218**	**100**%	**32,306**	**37.9**%
Nursing staff	56,693	66.5%	16,888	29.8%
Physicians total	28,525	33.5%	15,418	54.1%
***Including***				
Management personnel	966	1.1%	N/A	
Hospital epidemiologists	756	0.9%	N/A	
Clinical physicians	26,803	31.5%	15,418	57.5%
***Including***				
Primary care physicians	6468	7.6%	5672	87.7%
Pediatricians	5731	6.7%	4812	84.0%
Specialized doctors	14,604	17.1%	4934	33.8%
***Including***				
Gynecologists (obstetrician-gynecologists)	1916	2.2%	1328	69.3%
Neurologists	1303	1.5%	503	38.6%
Cardiologists	638	0.7%	292	45.8%
Infectious disease specialists	585	0.7%	217	37.1%
Endocrinologists	473	0.6%	268	56.7%
Allergists and immunologists	178	0.2%	137	77.0%
Pulmonologists	169	0.2%	119	70.4%
Nephrologists	105	0.1%	59	56.2%
Hematologists	82	0.1%	50	61.0%
Other clinical physicians (surgeons, urologists, otolaryngologists etc.)	9155	10.7%	1810	19.8%

## Data Availability

The data presented in this study are available on request from the corresponding author. The data are not publicly available due to the published data including only part of the work. The database is being processed on another topic and is being prepared for publication.

## Appendix A

**Table A1:** Proportion of healthcare workers ready to be vaccinated against COVID-19.

Gender		Ready to be Vaccinated	*p* < 0.001
	Male	3689 (43.2%; 42.2–44.3)	
	Female	26,673 (33.8%; 33.5–34.2)	
**Age**			*p* < 0.001
	20–30 years	4331 (24.2%; 23.6–24.9)	
	31–40 years	6741 (31%; 30.4–31.6)	
	41–50 years	9021 (37%; 36.4–37.6)	
	51–60 years	7422 (43.3%; 42.6–44.1)	
	61–70 years	2678 (45.8%; 44.5–47.1)	
**Work experience**			***p* < 0.001**
	Less than a year	1453 (24.9%; 23.8–26)	
	2–10 years	6148 (27.6%; 27–28.2)	
	11–20 years	7022 (33.4%; 32.7–34)	
	21–30 years	8100 (39.2%; 38.5–39.8)	
	31–40 years	6047 (43.9%; 43.1–44.8)	
	41–50 years	1525 (43.1%; 41.4–44.7)	
	>50 years	67 (40.9%; 33.3–48.4)	
**Place of work**			***p* < 0.001**
Outpatient polyclinic institutions		15,766 (37.4%; 36.9–37.9)	
Inpatient polyclinic institutions		10,317 (32.1%; 31.6–32.6)	
Dispensaries		1590 (34.7%; 33.3–36)	
Women’s consultations, Maternity hospitals		1307 (35%; 33.5–36.5)	
Ambulance and emergency care facilities		1171 (28.3%; 26.9–29.6)	
Spa facilities, preventive healthcare facilities		211 (36.5%; 32.6–40.4)	
**Healthcare facility in**			***p* < 0.001**
	Urban area	25,572 (33.9%; 33.5–34.2)	
	Rural area	4790 (40.5%; 39.6–41.4)	
**Federal district**			***p* < 0.001**
	Central FD	8354 (33.5%; 32.9–34.1)	
	Northwestern FD	4308 (32.7%; 31.9–33.5)	
	Volga FD	4643 (36.2%; 35.3–37)	
	Southern FD	1058 (38%; 36.2–39.8)	
	North Caucasian FD	970 (24.4%; 23.1–25.8)	
	Ural FD	2536 (36.6%; 35.5–37.8)	
	Siberian FD	4911 (33.7%; 32.9–34.5)	
	Far Eastern FD	3580 (44%; 42.9–45.1)	
**Involved in vaccination process**			***p* < 0.001**
	No	16,150 (30.5%; 30.1–30.9)	
	Yes	13,655 (42.3%; 41.7–42.8)	
**Vaccination attitude**			***p* < 0.001**
	Negative	989 (7.1%; 6.6–7.5)	
	Neutral	3946 (19%; 18.4–19.5)	
	Positive	24,870 (49.3%; 48.9–49.8)	

**Table A2:** Proportion of healthcare workers ready to be vaccinated against COVID-19 (depending on involvement in vaccination process).

	Total	Involved	Not Involved
**Total**	29,805 (35%; 34.7–35.3)	13,655 (42.3%; 41.7–42.8)	16,150 (30.5%; 30.1–30.9)
Nursing staff	17,723 (31.3%; 30.9–31.6)	6623 (39.2%; 38.5–40)	11,100 (27.9%; 27.4–28.3)
Physicians total	12,082 (42.4%; 41.8–42.9)	7032 (45.6%; 44.8–46.4)	5050 (38.5%; 37.7–39.4)
***Including***			
Management personnel	2604 (40.3%; 39.1–41.5)	2424 (42.7%; 41.4–44)	180 (22.6%; 19.7–25.5)
Epidemiologists	2513 (43.8%; 42.6–45.1)	2163 (45%; 43.5–46.4)	350 (38.1%; 34.9–41.2)
Clinical physicians	5170 (40.7%; 39.9–41.6)	1803 (50%; 48.4–51.6)	3367 (37.1%; 36.1–38.1)
***Including***			
Primary care physicians	783 (40.9%; 38.7–43.1)	642 (48.3%; 45.7–51)	141 (24%; 20.5–27.4)
Pediatricians	489 (37.5%; 34.9–40.2)	239 (47.5%; 43.2–51.9)	250 (31.3%; 28–34.5)
Specialized doctors	252 (39.5%; 35.7–43.3)	138 (47.3%; 41.5–53)	114 (32.9%; 28–37.9)
***Including***	348 (59.5%; 55.5–63.5)	226 (61.4%; 56.4–66.4)	122 (56.2%; 49.6–62.8)
Gynecologists (obstetrician-gynecologists)	203 (42.9%; 38.5–47.4)	128 (47.8%; 41.8–53.7)	75 (36.6%; 30–43.2)
Neurologists	75 (42.1%; 34.9–49.4)	65 (47.4%; 39.1–55.8)	10 (24.4%; 11.2–37.5)
Cardiologists	73 (43.2%; 35.7–50.7)	57 (47.9%; 38.9–56.9)	16 (32%; 19.1–44.9)
Infectious disease specialists	43 (41%; 31.5–50.4)	31 (52.5%; 39.8–65.3)	12 (26.1%; 13.4–38.8)
Endocrinologists	36 (43.9%; 33.2–54.6)	25 (50%; 36.1–63.9)	11 (34.4%; 17.9–50.8)
Allergists and immunologists	3651 (39.9%; 38.9–40.9)	894 (49.4%; 47.1–51.7)	2757 (37.5%; 36.4–38.6)
Pulmonologists	73 (43.2%; 35.7–50.7)	57 (47.9%; 38.9–56.9)	16 (32%; 19.1–44.9)
Nephrologists	43 (41%; 31.5–50.4)	31 (52.5%; 39.8–65.3)	12 (26.1%; 13.4–38.8)
Hematologists	36 (43.9%; 33.2–54.6)	25 (50%; 36.1–63.9)	11 (34.4%; 17.9–50.8)
Other clinical physicians (surgeons, urologists, otolaryngologists etc.)	3651 (39.9%; 38.9–40.9)	894 (49.4%; 47.1–51.7)	2757 (37.5%; 36.4–38.6)

**Table A3:** Question: “Are you ready to recommend vaccination against COVID-19?”—absolute value, proportion and 95% confidence interval.

Total		Yes	No	Not Sure	*p* < 0.001
**Gender**					
	Male	3241 (38.9%; 37.9–40)	2716 (32.6%; 31.6–33.6)	2365 (28.4%; 27.4–29.4)	
	Female	21,780 (28.3%; 28–28.6)	26,093 (33.9%; 33.6–34.3)	29,023 (37.7%; 37.4–38.1)	
**Age**					***p* < 0.001**
	20–30 years	3272 (18.6%; 18.1–19.2)	7963 (45.4%; 44.6–46.1)	6313 (36%; 35.3–36.7)	
	31–40 years	5242 (24.8%; 24.2–25.4)	8159 (38.6%; 38–39.3)	7724 (36.6%; 35.9–37.2)	
	41–50 years	7278 (30.6%; 30–31.2)	7505 (31.6%; 31–32.2)	8996 (37.8%; 37.2–38.4)	
	51–60 years	6371 (38.2%; 37.5–38.9)	3954 (23.7%; 23.1–24.3)	6356 (38.1%; 37.4–38.8)	
	61–70 years	2679 (46.9%; 45.6–48.2)	1143 (20%; 19–21.1)	1887 (33.1%; 31.8–34.3)	
**Work experience**					***p* < 0.001**
	Less than a year	1032 (18.3%; 17.3–19.3)	2331 (41.3%; 40–42.5)	2286 (40.5%; 39.2–41.7)	
	2–10 years	4741 (22.1%; 21.5–22.7)	9132 (42.6%; 41.9–43.2)	7576 (35.3%; 34.7–36)	
	11–20 years	5538 (27%; 26.4–27.6)	7433 (36.2%; 35.6–36.9)	7557 (36.8%; 36.2–37.5)	
	21–30 years	6723 (33.1%; 32.4–33.7)	5965 (29.3%; 28.7–30)	7652 (37.6%; 37–38.3)	
	31–40 years	5385 (39.6%; 38.8–40.4)	3168 (23.3%; 22.6–24)	5044 (37.1%; 36.3–37.9)	
	41–50 years	1532 (43.8%; 42.2–45.5)	737 (21.1%; 19.7–22.4)	1226 (35.1%; 33.5–36.7)	
	>50 years	70 (43.8%; 36.1–51.4)	43 (26.9%; 20–33.7)	47 (29.4%; 22.3–36.4)	
**Place of work**					***p* < 0.001**
Outpatient polyclinic institutions		13,431 (32.5%; 32.1–33)	11,843 (28.7%; 28.2–29.1)	16,042 (38.8%; 38.4–39.3)	
Inpatient polyclinic institutions		8248 (26.4%; 25.9–26.9)	11,965 (38.3%; 37.8–38.9)	10,997 (35.2%; 34.7–35.8)	
Dispensaries		1233 (28%; 26.7–29.3)	1664 (37.8%; 36.4–39.2)	1507 (34.2%; 32.8–35.6)	
Women’s consultations, Maternity hospitals		1007 (27.6%; 26.2–29.1)	1302 (35.7%; 34.1–37.3)	1338 (36.7%; 35.1–38.3)	
Ambulance and emergency care facilities		947 (23.1%; 21.8–24.4)	1851 (45.1%; 43.6–46.7)	1302 (31.8%; 30.3–33.2)	
Spa facilities, preventive healthcare facilities		155 (28.7%; 24.8–32.5)	184 (34%; 30–38)	202 (37.3%; 33.3–41.4)	
**Healthcare facility in**					***p* < 0.001**
	Urban area	20,997 (28.5%; 28.2–28.8)	25,475 (34.6%; 34.2–34.9)	27,222 (36.9%; 36.6–37.3)	
	Rural area	4024 (34.9%; 34–35.8)	3334 (28.9%; 28.1–29.8)	4166 (36.2%; 35.3–37)	
**Federal district**					***p* < 0.001**
	Central FD	7202 (29.6%; 29–30.2)	8086 (33.2%; 32.6–33.8)	9059 (37.2%; 36.6–37.8)	
	Northwestern FD	3450 (26.7%; 26–27.5)	4643 (36%; 35.2–36.8)	4810 (37.3%; 36.4–38.1)	
	Volga FD	3915 (31.1%; 30.2–31.9)	3993 (31.7%; 30.9–32.5)	4698 (37.3%; 36.4–38.1)	
	Southern FD	879 (32.1%; 30.4–33.9)	870 (31.8%; 30.1–33.5)	987 (36.1%; 34.3–37.9)	
	North Caucasian FD	832 (21.3%; 20–22.6)	1624 (41.6%; 40.1–43.2)	1446 (37.1%; 35.5–38.6)	
	Ural FD	1998 (29.8%; 28.8–30.9)	2171 (32.4%; 31.3–33.6)	2525 (37.7%; 36.6–38.9)	
	Siberian FD	3969 (27.9%; 27.2–28.7)	5294 (37.2%; 36.5–38)	4951 (34.8%; 34–35.6)	
	Far Eastern FD	2774 (35.5%; 34.4–36.6)	2128 (27.2%; 26.2–28.2)	2912 (37.3%; 36.2–38.3)	
**Involved in vaccination process**					***p* < 0.001**
	No	12,891 (24.4%; 24–24.7)	20,844 (39.4%; 39–39.8)	19,177 (36.2%; 35.8–36.7)	
	Yes	12,130 (37.5%; 37–38.1)	7965 (24.7%; 24.2–25.1)	12,211 (37.8%; 37.3–38.3)	
**Vaccination attitude**					***p* < 0.001**
	Negative	814 (5.8%; 5.4–6.2)	9838 (70.3%; 69.6–71.1)	3335 (23.8%; 23.1–24.5)	
	Neutral	2711 (13%; 12.6–13.5)	8432 (40.5%; 39.8–41.2)	9669 (46.5%; 45.8–47.1)	
	Positive	21,496 (42.6%; 42.2–43.1)	10,539 (20.9%; 20.5–21.3)	18,384 (36.5%; 36–36.9)	

**Table A4:** Question: “Are you ready to recommend vaccination against COVID-19?” (specialty). Absolute value, proportion and 95% confidence interval. *p* < 0.001.

Total	Yes	No	Not Sure
	25,021 (29.4%; 29.1–29.7)	28,809 (33.8%; 33.5–34.1)	31,388 (36.8%; 36.5–37.2)
Nursing staff	14,029 (24.7%; 24.4–25.1)	21,206 (37.4%; 37–37.8)	21,458 (37.8%; 37.5–38.2)
Physicians total	10,992 (38.5%; 38–39.1)	7603 (26.7%; 26.1–27.2)	9930 (34.8%; 34.3–35.4)
***Including***			
Management personnel	551 (57%; 53.9–60.2)	193 (20%; 17.5–22.5)	222 (23%; 20.3–25.6)
Epidemiologists	379 (50.1%; 46.6–53.7)	138 (18.3%; 15.5–21)	239 (31.6%; 28.3–34.9)
Clinical physicians	10,062 (37.5%; 37–38.1)	7272 (27.1%; 26.6–27.7)	9469 (35.3%; 34.8–35.9)
***Including***			
Primary care physicians	2562 (39.6%; 38.4–40.8)	1677 (25.9%; 24.9–27)	2229 (34.5%; 33.3–35.6)
Pediatricians	2225 (38.8%; 37.6–40.1)	1209 (21.1%; 20–22.2)	2297 (40.1%; 38.8–41.3)
Specialized doctors	4558 (35.9%; 35.1–36.8)	3833 (30.2%; 29.4–31)	4297 (33.9%; 33–34.7)
***Including***			
Gynecologists (obstetrician-gynecologists)	717 (37.4%; 35.3–39.6)	553 (28.9%; 26.8–30.9)	646 (33.7%; 31.6–35.8)
Neurologists	468 (35.9%; 33.3–38.5)	397 (30.5%; 28–33)	438 (33.6%; 31–36.2)
Cardiologists	241 (37.8%; 34–41.5)	184 (28.8%; 25.3–32.4)	213 (33.4%; 29.7–37)
Infectious disease specialists	338 (57.8%; 53.8–61.8)	113 (19.3%; 16.1–22.5)	134 (22.9%; 19.5–26.3)
Endocrinologists	180 (38.1%; 33.7–42.4)	113 (23.9%; 20–27.7)	180 (38.1%; 33.7–42.4)
Allergists and immunologists	74 (41.6%; 34.3–48.8)	49 (27.5%; 21–34.1)	55 (30.9%; 24.1–37.7)
Pulmonologists	72 (42.6%; 35.1–50.1)	40 (23.7%; 17.3–30.1)	57 (33.7%; 26.6–40.9)
Nephrologists	41 (39%; 29.7–48.4)	28 (26.7%; 18.2–35.1)	36 (34.3%; 25.2–43.4)
Hematologists	30 (36.6%; 26.2–47)	22 (26.8%; 17.2–36.4)	30 (36.6%; 26.2–47)
Other clinical physicians (surgeons, urologists, otolaryngologists etc.)	3114 (34%; 33–35)	2887 (31.5%; 30.6–32.5)	3154 (34.5%; 33.5–35.4)

**Table A5:** Proportion of healthcare workers, ready to be vaccinated against COVID-19 (depending on the age of physicians and nursing staff).

Age, Years	Physicians Total	Nursing Staff
20–30	1901 (29.3%; 27.3–31.3)	2346 (21.2%; 19.5–22.9)
31–40	2919 (38.9%; 37.1–40.7)	3687 (27.1%; 25.7–28.5)
41–50	2837 (46.9%; 45.1–48.7)	6032 (34.0%; 32.8–35.2)
51–60	2982 (52.4%; 50.6–54.2)	4299 (39.1%; 37.6–40.6)
61–70	1336 (52.1%; 49.4–54.8)	1302 (41.4%; 38.7–44.1)

**Table A6:** Question: “Are you ready to recommend vaccination against COVID-19?” depending on the age of physicians and nursing staff.

Age, Years	Physicians Total	Nursing Staff
20–30	1562 (15.5%; 13.7–17.3)	1710 (24%; 22.0–26.0)
31–40	2497 (20.1%; 18.5–21.7)	2745 (33.3%; 31.5–35.1)
41–50	2585 (26.5%; 24.8–28.2)	4693 (42.8%; 41.4–44.2)
51–60	2811 (32.4%; 30.7–34.1)	3560 (49.4%; 47.8–51)
61–70	1415 (40.2%; 37.6–42.8)	1264 (55.2%; 52.5–57.9)
